# Commentary: Scalar diversity, negative strengthening, and adjectival semantics

**DOI:** 10.3389/fpsyg.2025.1657242

**Published:** 2025-10-07

**Authors:** Nadra Salman, Amit Almor

**Affiliations:** ^1^Linguistics Program, University of South Carolina, Columbia, SC, United States; ^2^Psychology Department, University of South Carolina, Columbia, SC, United States; ^3^Institute of Mind and Brain, University of South Carolina, Columbia, SC, United States

**Keywords:** affect, arousal, scalar implicature, negative strengthening, gradable adjectives

## 1 Introduction

Scalar implicatures (SIs) and negative strengthening (NS) are two central pragmatic inferences licensed by adjectives. SIs arise when the use of a weaker term (e.g., *content*) implies that its stronger scale mate (e.g., *happy*) does not apply, whereas NS occurs when the negation of a stronger term implies that the negation of its weaker scale mate does not apply (e.g., *unhappy* meaning “not content”).

Early work demonstrated that some scalar terms systematically yield lower SI rates than others (Doran et al., 2009), and later large-scale studies confirmed that this diversity is robust across a broad range of adjectives and quantifiers (van Tiel et al., 2016). While previous studies have demonstrated various sources of this SI derivation like properties of the scale and the scale mates, recently NS has been identified as a factor that also affects SI derivation rates (Benz et al., 2018). On this account, judgments about weaker terms such as *good* involve a competition between a scalar implicature (“not excellent”) and a negatively strengthened interpretation of the stronger term.

Extending this work, (Gotzner et al. 2018a; henceforth GSB, see also Gotzner et al., 2018b) investigated a larger set of adjectives and confirmed the negative correlation between SIs and NS. They explain this trade-off partly through a blocking mechanism, whereby the strengthened interpretation of a negated strong adjective (e.g., *not stunning* → “rather ugly”) can conflict with, and therefore reduce, endorsement of the scalar implicature derived from its weaker scale-mate (e.g., *attractive* → “attractive but not stunning”). They also systematically coded semantic properties of adjectives and adjectival scales (e.g., extremeness and boundedness) and demonstrated that these properties also predict the rate of SIs and NS.

More recently, (Gotzner and Mazzarella 2024) demonstrated that NS is sensitive to evaluative polarity beyond structural properties. Testing absolute adjectives in the *not very* construction, they found that evaluatively positive adjectives (*clean, closed*) are more likely to be strengthened than evaluatively negative ones (*dirty, open*), with effects confirmed using valence ratings from emotion lexicons. Their findings reveal that both evaluative polarity and scale structure modulate negative strengthening, indicating that affective meaning directly shapes pragmatic inference.

Building on this, we ask whether the variability in SI and NS rates that has been attributed to structural features and the anticorrelation between the two inference types could also depend on broader cognitive factors. Many of the adjectives GSB identified as structurally extreme (e.g., *stunning, terrible, or perfect*) are also affectively salient, suggesting that arousal and valence could underlie some of the same patterns previously attributed to scale structure alone. This overlap raises the possibility that that the observed tradeoff between SIs and NS reflects differences in affective activation as much as lexical scale structure on top of the other properties explored by GBS. Arousal is central to conceptual semantic representations and influences attention and language processing (Citron, 2012; Kensinger and Schacter, 2008), making it a plausible factor in the derivation of scalar inferences. Despite its potential relevance, the role of affect in pragmatic inference has not been systematically examined (cf. see Alexandropoulou and Gotzner, 2024, on evaluative polarity's role in NS).

The present study therefore tests whether arousal and valence predict variation in SI and NS endorsement for adjectives beyond what is accounted for by structural features. Specifically, we investigate two questions: first, whether affective properties of adjectives contribute additional explanatory power for predicting scalar inference rates, and second, whether the strength of the observed anticorrelation between SIs and NS varies as a function of affective salience. By systematically examining these affective dimensions alongside established structural predictors, we aim to determine whether incorporating psychological factors provides a more complete account of the variability observed in pragmatic inference patterns.

## 2 Methods

We reanalyzed the dataset from GSB using stepwise regression, incorporating corpus affective ratings from (Warriner et al. 2013). The models tested whether arousal and valence predicted SI and NS endorsement beyond structural predictors identified in prior work (e.g., boundedness and polarity). Of the 45 adjective pairs in the original study, 8 lacked affective ratings and were excluded, yielding a final sample of 37 pairs. Of the original 45 initial pairs, 8 were excluded due to missing affective ratings, leaving a final sample of 37 pairs.

## 3 Results

**Figure 1:**
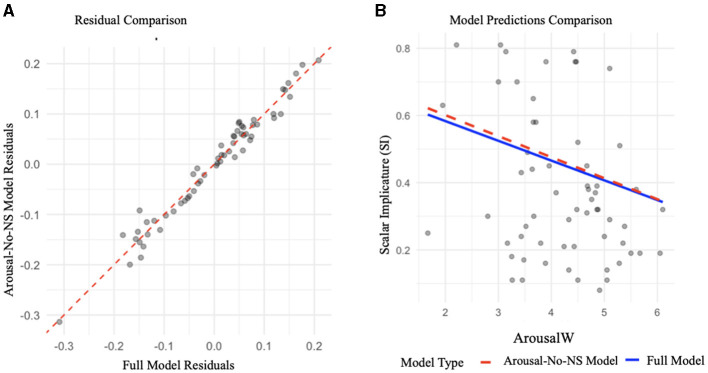
**(A)** Comparison of residuals from the Full Model and the Arousal-No-NS Model, with alignment along the 45-degree line indicating similar fits. **(B)** Relationship between arousal ratings and scalar implicature (SI). Both the Full Model (blue line) and the Arousal-No-NS Model (red dashed line) predict a consistent negative association, showing that excluding negative strengthening (NS) does not alter the overall effect of arousal.

Consistent with GSB, SI and NS were negatively correlated (*r* = −0.67, *p* < 0.001). SI was also negatively correlated with the arousal of the weaker adjective (ArousalW; *r*= −0.347*, p* = 0.015). When ArousalW was included in the in the full model predicting SI, the coefficient for NS was substantially reduced and no longer a significant predictor (β = −0.184, *p* = 0.298), and adding NS to the full model with ArousalW did not improve predictive power [*F*_(1)_=1.12, *p* = 0.298] (see Figure 1).

This pattern held across partial models, suggesting that ArousalW and NS share overlapping predictive variance for SI rates, with ArousalW accounting for much of the relationship previously attributed to NS alone. The arousal of the stronger adjective was only marginally significant in the full model, while the valence of both the weak and strong adjectives were not significant predictors, suggesting minimal impact of valence on SI derivation. Structural predictors, including semantic distance, extremeness, and polarity, remained robust predictors of SI in all models. In contrast, For NS, inference patterns were primarily explained by structural features (semantic distance, extremeness, boundedness), with negligible contributions from affective dimensions.

## 4 Discussion

Our findings confirm the negative correlation between SI and NS reported by GSB but also show that this relationship can be explained by the affective salience of the weaker adjective licensing the implicature. Once arousal was included as a predictor, NS no longer accounted for variance in SI endorsement. This suggests that the apparent competition between SI and NS arises not from a direct pragmatic conflict, but from the way highly arousing weaker adjectives capture attention and increase processing effort, reducing the likelihood of implicature derivation.

This explanation contrasts with GSB's blocking account, in which the strengthened interpretation of a negated strong adjective conflicts with the implicature of the weak term, creating a structural tradeoff. Our results suggest that this tradeoff can also emerge indirectly, as a byproduct of affective activation. This pattern suggests that affective activation may influence scalar inference rates through the processing demands associated with the weaker adjective that triggers the implicature.

Situating this within broader processing research that attributes variability in scalar inference to individual differences and processing cost shaped by structural and contextual factors (see Khorsheed et al., 2022; Khorsheed and Gotzner, 2023) highlights its theoretical significance. (Antoniou et al. 2016) demonstrated that individual differences in working memory capacity and age predict scalar implicature derivation, with higher working memory and younger age associated with greater implicature rates. They attributed this variation to the processing demands of coordinating multiple information sources and maintaining speaker knowledge states during pragmatic inference.

The task design used by GBS may partially explain why arousal effects were specific to the weaker adjective: participants first processed statements like “James is popular” and then determined whether this implies “James is not famous,” making the arousal properties of the initially encountered weaker adjective more cognitively relevant to the task than the stronger term mentioned only in the response options. However, another possible and not mutually exclusive explanation is that speakers often use weaker terms for indirect communication and conflict avoidance (Pinker et al., 2008; Gotzner and Scontras, 2024). High arousal in weak adjectives may create tension between the speaker's apparent preference for indirectness and the emotional intensity of the term itself, potentially disrupting the cognitive processes underlying implicature derivation. Crucially, research on emotional processing demonstrates that arousal places significant demands on working memory and cognitive resources, with highly arousing stimuli competing for limited attentional capacity and disrupting working memory performance (see Hou and Cai, 2022). Our findings extend this cognitive resources framework by identifying affective salience as an additional factor that may influence the processing costs associated with scalar inference derivation, potentially explaining why some adjectives show different implicature patterns despite similar structural properties.

Overall, our findings demonstrate that affective properties of adjectives contribute systematically to scalar inference patterns beyond established structural predictors. By identifying arousal as a factor that influences the apparent SI-NS relationship, this work highlights the importance of considering psychological salience alongside lexical semantics in models of pragmatic reasoning.
